# Implementing an audit and feedback cycle to improve adherence to the Choosing Wisely Canada recommendations: clustered randomized trail

**DOI:** 10.1186/s12875-022-01912-7

**Published:** 2022-11-26

**Authors:** Alexander Singer, Leanne Kosowan, Elissa M. Abrams, Alan Katz, Lisa Lix, Katrina Leong, Allison Paige

**Affiliations:** 1 Department of Family Medicine, Rady Faculty of Health Sciences, University of Manitoba, D009-780 Bannatyne Ave, Winnipeg, MB R3E0W2 Canada; 2 Department of Pediatrics, Section of Allergy and Clinical Immunology, Rady Faculty of Health Sciences, University of Manitoba, Winnipeg, MB Canada; 3 Department of Pediatrics, Division of Allergy and Immunology, University of British Columbia, Vancouver, BC Canada; 4 Departments of Community Health Science & Family Medicine, Rady Faculty of Health Sciences, University of Manitoba, Winnipeg, MB Canada; 5 Manitoba Centre for Health Policy, Winnipeg, MB Canada; 6 Department of Community Health Sciences, Rady Faculty of Health Sciences, University of Manitoba, Winnipeg, MB Canada

**Keywords:** Primary health care, Health care quality, Access and evaluation, Health services research, Choosing wisely, Randomized controlled trial

## Abstract

**Background:**

Audit and Feedback (A&F), a strategy aimed at promoting modified practice through performance feedback, is a method to change provider behaviour and reduce unnecessary medical services. This study aims to assess the use of A&F to reduce antibiotic prescribing for viral infections and antipsychotic prescribing to patients with dementia.

**Methods:**

Clustered randomized trial of 239 primary care providers in Manitoba, Canada, participating in the Manitoba Primary Care Research Network. Forty-six practices were randomly assigned to one of three groups: control group, intervention 1 (recommendations summary), intervention 2 (recommendations summary and personalized feedback). We assessed prescribing rates prior to the intervention (2014/15), during and immediately after the intervention (2016/17) and following the intervention (2018/19). Physician characteristics were assessed.

**Results:**

Between 2014/15–2016/17, 91.6% of providers in intervention group 1 and 95.9% of providers in intervention group 2 reduced their antibiotic and antipsychotic prescribing rate by ≥ 1 compared to the control group (77.6%) (*p*-value 0.0073). This reduction was maintained into 2018/19 at 91.4%. On multivariate regression alternatively funded providers had 2.4 × higher odds of reducing their antibiotic prescribing rate compared to fee-for-service providers. In quantile regression of providers with a reduction in antibiotic prescribing, alternatively funded (e.g. salaried or locum) providers compared to fee-for-service providers were significant at the 80^th^ quantile.

**Conclusions:**

Both A&F and recommendation summaries sent to providers by a trusted source reduced unnecessary prescriptions. Our findings support further scale up of efforts to engage with primary care practices to improve care with A&F.

**Trial registration:**

ClinicalTrials.gov NCT05385445, retrospectively registered, 23/05/2022.

**Supplementary Information:**

The online version contains supplementary material available at 10.1186/s12875-022-01912-7.

## Background

Reducing overuse of medical services is increasingly being recognized as an international public health priority [1]. The Canadian Institute for Health Information demonstrated that ~ 30% of Canadians had unnecessary diagnostic investigations and treatments [2]. A recent survey of physicians in the United States reported that 20% of overall medical care was unnecessary, including 25% of diagnostic tests, 20% of prescriptions and 10% of procedures [3]. An Institute of Medicine report touted unnecessary services as the biggest contributor to financial waste in the United States, resulting in $210 billion in unnecessary spending [3]. Global consumption of antibiotic medications have increase by 36% between 2000 and 2010 largely attributed to increased use in Brazil, China, India, Russia and South Africa [1]. Further to this, the misuse and abuse of antimicrobial medication has accelerated global rates of antimicrobial resistance [4]. The European Centre for Disease Control estimates that antimicrobial resistance causes 25,000 deaths each year and approximately 2.5 million extra hospital days [4]. Overprescribing and over-investigation is an ongoing issue, particularly in the area of antimicrobials [5–7].

Choosing Wisely Canada (CWC), launched in 2014, is part of a movement to improve Canadian healthcare quality. Choosing Wisely engages healthcare providers and patients in shared decision-making for medically necessary, evidence-based, and safe care to reduce medical overuse in healthcare settings [8]. CWC has partnered with national and provincial medical associations to create recommendations with the goal of reducing harm through judicious use of medical testing and interventions. Several CWC recommendations suggest the avoidance of antimicrobials for viral infections owing to the risk of adverse events and promotion of antibiotic resistance [9–14]. Additionally, CWC recommends against the use of antipsychotics in patients with dementia given the evidence of increased mortality [15–19].

Several methods have been proposed to systematically study and reduce the burden of unnecessary care including public campaigns and reporting, tailoring laboratory requisitions and prescribing practices, health technology and promotion of evidence-based guidelines [20]. One of the most consistently used methods are Audit and Feedback (A&F) interventions. In healthcare, A&F is a strategy wherein physicians receive performance feedback that demonstrates differences between actual and desired behaviours when compared to evidence-based guidelines [20–22]. The effects of A&F on physician behaviour are variable. Some studies report a small effect [21, 23], while others demonstrate substantial changes in clinical practice [24, 25]. A 2012 Cochrane review found that delivery of feedback is more effective when baseline performance is low, it is provided by a colleague or supervisor, written and verbal formats are available, and it is delivered in multiple sessions with explicit targets and an action plan [26]. Overall, evidence suggests that A&F has moderate impact on prescribing and treating behaviours [21, 23–27]. Though many studies have looked at process and design factors that impact A&F response, there is a lack of research that considers which physician factors affect the likelihood of responding to this strategy, particularly within a Canadian context.

The objectives of this study were: (1) to test the use of practice-specific A&F to reduce antibiotic prescribing for viral infections and antipsychotic prescribing to patients with dementia, (2) to assess which factors influence the likelihood of responding to A&F, and (3) to determine if desired practice changes were stable two-years after the intervention.

## Methods

We conducted a clustered randomized trial of primary care providers in Manitoba, Canada participating in the Manitoba Primary Care Research Network (MaPCReN) to assess the impact of A&F interventions to reduce potentially unnecessary prescribing.

MaPCReN is Manitoba’s provincial network from the Canadian Primary Care Sentinel Surveillance Network (CPCSSN). CPCSSN is the largest multi-system database in Canada to extract and process de-identified data from consenting primary care clinician electronic medical records (EMR) to build a pan-Canadian EMR-based repository for health surveillance, research, and quality improvement. For this study we used the patient, billing, encounter diagnosis, problem list, prescribed medication, laboratory results and provider tables from the MaPCReN repository.

### Description of the intervention

In January 2016, MaPCReN included 239 providers in 46 practices across Manitoba, representing ~ 20% of Manitoba primary care providers. MaPCReN recruits consenting primary care providers (family physicians, nurse practitioners and community pediatricians) that receive biannual feedback reports for practice reflection and quality improvement. Feedback reports offer provider-specific practice-level details including disease prevalence and clinical characteristics of patients, reported in comparison to other providers at the same clinic, health region and provincially (Additional file 1 Appendix A). There were 61 providers who changed EMRs, changed practices or left practices over the study period and were not included in the overall sample.

We implemented an A&F cycle reporting on practice behaviours related to CWC recommendations. Based on their prior consent to participate in feedback studies, all clinics participating in MaPCReN were randomized to one of three intervention groups. MaPCReN personnel used SAS V9.4 (SAS Institute Inc. Cary, NC) to generate a randomization allocation sequence that randomly assigned a number to clinics. Randomization at the clinic level prevented contamination that could be caused by two providers from the same practice randomized to different arms. Number of physicians practicing at each clinic was controlled during randomization. The intervention was initially designed to address four CWC recommendations. However, the two recommendations related to laboratory testing were not included in our final analysis due to changes in provincial laboratory ordering requisitions and digitization of reporting protocols that were implemented during the study period, affecting the reliability and quality of laboratory testing data.

We focused our analysis on two CWC recommendations:

- ➣ Don’t use antibiotics for upper respiratory infections that are likely viral in origin, such as influenza-like illness, or self-limiting, such as sinus infections of less than seven days of duration [9–14].
- ➣ Don't use antipsychotics as a first choice to treat behavioural and psychological symptoms of dementia [15–19].

Based on literature review and expertise of our team, this study assessed two intervention groups compared to a control group [26–30]. It has been suggested that A&F may be more effective when provided in multiple forms [26] additionally research has not assessed the impact of type of feedback (i.e. provider-specific information vs. background information). The intervention “Group 1” received the standard feedback report (Additional file 1 Appendix A) with a one-page summary of the CWC recommendations of interest (Additional file 1 Appendix B). They did not receive any data specific to their prescribing. Intervention “Group 2” received the standard feedback report (Additional file 1Appendix A), CWC recommendation summary (Additional file 1 Appendix B) and practice-specific data related to their prescribing rates for the CWC recommendations of interest, compared to rates for other providers at their clinic, in their health region and in the province (Additional file 1 Appendix C). The control group received the standard feedback report with no information related to CWC (Additional file 1  Appendix A).

The A&F intervention took place between January 2016 and January 2017. Participating providers were emailed two feedback reports during the intervention period (January 2016 and July 2016). Retrospective EMR data from the MaPCReN repository was assessed between January 2014-December 2019 to assess changes in prescribing rates during and immediately following the A&F intervention (2016/17), as well as assess continued change in practice after the A&F (2018/19) compared to prescribing rates prior to the A&F (2014/15). There were seven clinics not included in the 2018/19 analysis due to a change in EMR vendor that may have compromised data quality.

### Covariates

#### Prescribing practices

Among patients with an antibiotic prescription (Anatomical Therapeutic Chemical Classification System (ATC) code: starting with J01) we searched for an encounter diagnosis code representing a viral infection, within seven days of the prescription. We used the International Classification of Diseases (ICD-9-CM) codes: 461 (acute mild-to-moderate sinusitis), 465 (upper respiratory tract infection), 466 (bronchitis), 460 (acute rhinitis), 464 (acute laryngitis and tracheitis), 477 (nasopharyngitis) or 487/488 (influenza) to indicate a viral diagnosis. Among patients 65 years or older with a diagnosis of dementia (ICD-9-CM: 290, 294 and 331) we evaluated the presence of antipsychotic medications based on the presence of an ATC code starting with N05A.

#### Provider characteristics

Clinic postal code was used to determine rural or urban clinic location. Practice size represents the number of patients that are assigned to the provider. Medical school location was categorized as Canadian compared to international medical school graduation. Clinician type included family physician, or nurse practitioner. Funding model was categorized as fee-for service or alternative funding (i.e. salaried or capitation payment). Fee-for-service describe providers remunerated based on submitted tariff codes on a per service basis.

### Statistical analysis

We report descriptive analytics on the use of non-recommended prescribing of antibiotics for viral infections [9], and antipsychotics in patients with dementia [15]. We calculated the antibiotic prescribing rate by dividing the annual number of antibiotics prescribed to patients (prescriptions for ATC J01) with a viral indication in each practice by the annual number of patients in each practice with a visit for a viral infection. We calculated the antipsychotic prescribing rate by dividing the number of antipsychotic medications prescribed (prescriptions for ATC N05A) to a patient ≥ 65 years diagnosed with dementia in the providers practice by the number of patients ≥ 65 years diagnosed with dementia in each practice. Within each study group (i.e. intervention group 1, intervention group 2 and control group) we assessed the change in prescribing. We compared the groups for reduction in one or both of the prescribing practices of interest. Further to this we assessed the stability of the practice change following the intervention for each of our intervention/control groups.

The multivariable logistic model compared providers with a decrease in their antibiotic or antipsychotic prescribing rate by ≥ 1 (yes vs. no) and provider characteristics: (sex (male vs female), age (continuous), provider type (family physician vs. nurse practitioner), funding model (fee-for-service vs alternative funding), clinic location (rural vs urban), country of medical training (international vs. Canadian). Additional multivariable logistic models compared providers with a decrease in their prescribing rate ≥ 1 for each medication type using the same covariates. Regression models controlled for inclusion in the intervention (group 1 or group 2) vs. control group. We present the odds ratios (OR) and 95% confidence interval (CI).

Among providers with a reduction in their antibiotic and antipsychotic prescribing rates we used quantile regression to assess reductions at the median (50^th^ quantile) as well as the 20^th^ and 80^th^ quantiles using the same covariates as the logistic regression analysis. Statistical analyses were conducted using SAS V9.4 (SAS Institute Inc. Cary, NC).

### Ethics approval

The Health Research Ethics Board at the University of Manitoba approved this study.

## Results

There were 178 primary care providers that participated in MaPCReN between January 1, 2016, and December 31, 2019. In 2019, participating providers represented 226,775 patients. Table 1 describes the characteristics of the providers. Providers in the control group were not significantly different from provider in the intervention groups based on sex, age, practice size or provider type. However, providers within intervention group 1 were less likely to use a fee-for-service remuneration model (*p*-value 0.0007), and providers in intervention group 2 were less likely to have obtained their medical training in Canada (*p*-value 0.042) compared to the control group. Additionally, providers in intervention group 2 were significantly less likely to practice at an urban clinic compared to providers in intervention group 1 (*p*-value 0.0378) (Table 1).

**Table 1:** Characteristics of primary care providers participating in MaPCReN by randomized group

Characteristic	MaPCReN providers *N* = 178	Control Group *N* = 58	Intervention Group 1: Background Information *N* = 71	Intervention Group 2: Modified Report *N* = 49
Female vs male provider	104 (58.4%)	32 (55.2%)	46 (64.8%)	26 (53.1%)
Provider age, mean(SD)	45.3 (9.8)	46.1 (10.2)	45.5 (9.6)	44.0(9.8)
Practice size (2014/2015), mean(SD)	729 (511)	729 (605)	629 (350)	874 (559)
Family physician vs. nurse practitioner	159 (89.3%)	54 (93.1%)	59(83.1%)	46 (93.9)
Fee-For-Service vs salaried	81 (45.5)	32 (55.2%)	20 (28.2%)	29 (59.2%)
Canadian graduate vs. international graduate	131 (73.6%)	49 (84.5%)	51 (71.8%)	31 (63.3%)
Urban vs rural clinic	123 (69.1%)	39 (67.2%)	56 (78.9%)	28 (57.1%)

Between 2014/15 (prior to A&F) and 2016/17 (during and immediately following A&F), 91.6% of providers in intervention group 1 and 95.9% of providers in intervention group 2 decreased their prescribing rate by ≥ 1 antibiotics or antipsychotic prescription. This was significantly higher than the percent of providers in the control group that reduced their prescribing rate by ≥ 1 (77.6%) (*p*-value 0.0073). This reduction was maintained into 2018/19 with 91.4% of providers decreasing their antibiotic or antipsychotic prescribing rate by ≥ 1 when compared to their 2014/15 rate (i.e. 93.2% in intervention group 1, 95.5% in intervention group 2, 87.2% in the control group) (Table 2).

**Table 2:** Decrease in prescribing rate by medication type prior to A&F (2014/15), during (2016/17) and post-A&F (2018/19)

Time period	MaPCReN providers *N* = 178	Control *N* = 58	Group 1 N = 71	Group 2: *N* = 49	*P*-value		
					Control vs Group 1	Control vs Group 2	Group 1 vs Group 2
Number of providers with a decrease in antibiotic or antipsychotic prescribing rate ≥ 1 (n, % of providers)							
2014/15 vs 2016/17	157/178 (88.2%)	45/58 (77.6%)	65/71 (91.5%)	47/49 (95.9%)	**0.0026**	**0.0065**	**0.3456**
2014/15 vs 2018/19^a^	117/128 (91.4%)	41/47 (87.2%)	55/59 (93.2%)	21/22 (95.5%)	0.2949	0.2919	0.7102
Number of providers with a decrease in antibiotic prescribing rate by ≥ 1 (n, % of providers)							
2014/15 vs 2016/17	115/178 (64.6%)	35/58 (60.3%)	49/71 (69.0%)	31/49 (63.3%)	0.127	0.8437	0.2058
2014/15 vs 2018/19^a^	100/128 (78.1%)	35/47 (74.5%)	48/59 (81.4%)	17/22 (77.3%)	0.894	0.9141	0.1918
Number of providers with a decrease in antipsychotic prescribing rate by ≥ 1 (n, % of providers)							
2014/15 vs 2016/17	105/178 (59.0%)	25/58 (32.6%)	43/71 (60.6%)	37/49 (75.5%)	**0.0482**	**0.0007**	0.0878
2014/15 vs 2018/19^a^	70/128 (54.7%)	22/47 (36.7%)	33/59 (46.1%)	15/22 (68.2%)	0.3503	0.0971	0.3183

Bolding indicates the *p*-value was statistically significant

Group 1: Background information

Group 2: Modified report

^a^Providers at a clinic that changed EMR vender were not included in the 2018/19 results

In 2014/15 MaPCReN providers on average (median) prescribed 12 antibiotic medications (IQR 41.5) for a viral indication representing a prescription for on average 16.0% (SD15.8) of patients with a visit for a viral infection. In 2016/17 the median number of prescriptions was 9 (IQR 37) (average prescribing rate 9.3% (SD8.8)) and in 2018/19 the median was 3 medications (IQR 7) (average prescribing rate 5.1% (SD 5.7)). The largest decrease in antibiotic prescribing (2014/15–2016/17) was observed in intervention group 1 (17.9% to 7.8%). There was a further reduction in the post-intervention period (2018/19), with the greatest reduction observed in intervention 1 (17.9% vs 3.9%) (Table 3).

**Table 3:** Prescribing rates by providers in each of the intervention groups by medication type

Variable	MaPCReN providers *N* = 178	Control Group *N* = 58	Group 1: Background Information *N* = 71	Group 2: Modified Report *N* = 49
Antibiotic prescribing				
2014/15 antibiotic prescribing rate, %(SD)	16.0 (SD15.8)	15.4 (SD17.3)	17.9 (SD18.4)	14.1 (SD8.5)
2016/17 antibiotic prescribing rate, %(SD)	9.3 (SD8.8)	7.6 (SD6.5)	7.8 (SD9.6)	13.2 (SD9.0)
2018/19 antibiotic prescribing rate, %(SD)	5.1 (SD5.7)	6.2 (SD6.7)	3.9 (SD4.4)	5.7 (SD5.7)
Antipsychotic prescribing				
2014/15 antipsychotic prescribing rate, %(SD)	17.7 (SD16.7)	13.7 (SD15.7)	17.9 (SD16.6)	22.5 (SD17.2)
2016/17 antipsychotic prescribing rate, %(SD)	10.5 (SD22.7)	9.5 (SD19.7)	13.0 (SD23.8)	8.1 (SD24.8)
2018/19 antipsychotic prescribing rate, %(SD)	10.6 (SD31.6)	4.1 (12.7)	16.4 (SD37.5	10.4 (SD37.5)

Antibiotic prescribing rate: the number of antibiotic medications prescribed for a viral indication / number of patients in the practice

Antipsychotic prescribing rate: the number of antipsychotic medications prescribed to patients diagnosed with dementia/ number of patients in the practice diagnosed with dementia

In 2014/15 MaPCReN providers prescribed on average 3 antipsychotic medications (SD5.5) to patients living with dementia. Providers decreased their prescribing rate from 17.7% (SD16.7) in 2014/15 to 10.5% (SD22.7) in 2016/17 (Table 3). Intervention group 2 had the largest decrease in prescribing rates, 22.5% (SD17.2) in 2014/15 to 10.4% (SD37.5) in 2016/17(Table 3). This decrease in prescribing was maintained in 2018/19 with an average prescribing rate of 10.6% (SD31.6) (Table 3). Overall, providers in this study reduced their antipsychotic prescribing from an average of 3 (SD 4) prescriptions per provider in 2014/15 to 1 (SD3.7) prescription in 2018/19.

On multivariate analyses by medication type we found that fee-for-service providers were significantly less likely to decrease their prescribing of antibiotics for a viral indication compared to salaried providers (OR 2.47, CI 1.06–5.8). No provider characteristics were associated with a decrease in antipsychotic prescribing for patients living with dementia. Providers in the intervention group had significantly higher odds of a reduction in medication (OR 4.28, CI 1.56–11.76) (Table 4).

**Table 4:** Logistic regression of decrease in prescribing rate by ≥ 1 medication between 2014/15 and 2016/17

Variable	Antibiotics or Antipsychotic (OR, 95%CI)	Antibiotics (OR, 95%CI)	Antipsychotic (OR, 95%CI)
Male vs female provider	0.69 (0.25–1.95)	0.68 (0.34–1.37)	1.44 (0.71–2.9)
Provider age (per 1 year increase in age)	1.04 (0.98–1.09)	1.01 (0.98–1.05)	1.03 (0.99–1.06)
Family physician vs. nurse practitioner	1.4 (0.25–7.83)	1.86 (0.59–5.88)	1.06 (0.36–3.12)
Salaried vs. fee-for-service provider	1.59 (0.49–5.19)	**2.38 (1.01–5.6)**	0.57 (0.24–1.32)
Urban vs rural clinic	0.41 (0.11–1.51)	1.24 (0.51–258)	0.59 (0.24–1.46)
Canadian graduate vs. international graduate	1.24 (0.35–4.46)	1.15 (0.51–2.58)	0.68 (0.3–6.74)
Intervention group vs control group	**4.28 (1.56–11.76)**	1.36 (0.67–2.78)	**3.29 (1.6–6.74)**

*OR* odds ratio, *95%CI* 95% confidence interval

Among those that did reduce their prescribing there were factors associated with a larger reduction. Despite no significant difference in antibiotic prescribing rates between rural and urban clinics (*p*-value 0.8329) or related to funding model (*p*-value 0.6214) in 2014/15. At the 50^th^ and 80^th^ quantiles alternatively funded (e.g. salaried) providers were significant in reduction of antibiotic prescribing compared to fee-for-service providers. Additionally, at the 50^th^ and 80^th^ quantiles providers in an intervention group were significant compared to the control group (Table 5). When we looked at the antipsychotic prescribing reductions at the 50^th^ and 80^th^ quantiles no factors were significant. However, rural clinics were significant at the 20^th^ quantile when compared to urban clinics (Table 6).

**Table 5:** Quantile Regression of factors associated with reduction in antibiotic prescribing rate (2014/15–2016/17) *N* = 115

Variable	50^th^ Quantile (4.1%)		20^th^ Quantile (0.5%)		80^th^ quantile (23.5%)	
	β	95%CI	β	95%CI	β	95%CI
Male vs female provider	-0.005	-0.03, 0.025	-0.017	-0.105, 0.111	-0.005	-0.017, 0.003
Provider age (continuous)	0.000	-0.001, 0.001	0.002	-0.004, 0.005	0.000	-0.001, 0.000
Family physician vs nurse practitioner	-0.011	-0.057, 0.07	0.071	-0.132, 0.878	0.006	-0.005, 0.034
Salaried vs fee-for-service	**0.034**	**0.014, 0.066**	0.139	-0.016, 0.287	**-0.011**	**0.004, 0.022**
Rural vs. urban clinic	-0.03	-0.022, 0.044	0.028	-0.091, 0.137	-0.002	-0.008, 0.008
Canadian graduate vs international graduate	-0.05	-0.077, -0.005	-0.07	-0.307, 0.022	-0.011	-0.027, 0.000
Intervention vs control group	**0.038**	**0.000–0.082**	0.037	-0.04, 0.35	**0.016**	**0.002, 0.031**

Bold indicated significance at 0.05

**Table 6:** Quantile Regression of factors associated with reduction in antipsychotic prescribing rate (2014/15–2016/17) *N* = 105

Variable	50^th^ Quantile (17.1%)		20^th^ Quantile (7.7%)		80^th^ quantile (28.6%)	
	β	95%CI	β	95%CI	β	95%CI
Male vs female provider	-0.004	-0.059, 0.055	0.006	-0.028, 0.041	-0.017	-0.172, 0.075
Provider age (continuous)	-0.002	-0.004, 0.002	0.000	-0.002, 0.002	-0.002	-0.01, 0.005
Fee-for-Service vs. salaried	-0.062	-0.109, 0.048	0.005	-0.063, 0.038	-0.072	-0.18, 0.117
Rural vs. urban clinic	0.104	-0.011, 0.177	**0.03**	**0.013, 0.094**	0.128	-0.091, 0.404
Canadian graduate vs international graduate	-0.006	-0.073, 0.092	-0.014	-0.056, 0.036	-0.011	-0.091, 0.154
Intervention vs control group	-0.045	-0.096,0.012	-0.005	-0.102, 0.042	-0.045	-0.157, 0.083

Bold indicated significance at 0.05

## Discussion

Our study is the first in Canada to demonstrate that the use of extended feedback reports resulted in significant change in practice patterns, with a reduction in use of antibiotics to treat viral infections, and antipsychotic prescribing to patients with dementia. Both practice-specific and generic recommendations related to CWC recommendations demonstrated the ability to lead to long lasting practice change. In the intervention groups, the prescribing of an antibiotic to treat a viral indication decreased from a prescribing rate from 16.0% in 2014/15 to a prescribing rate of 10.5% in 2016/17. Importantly, reductions were maintained, and at times further reduced, in the subsequent two-year period after the intervention. Additionally, antipsychotic prescribing in patients with dementia decreased from a prescribing rate of 20.2% in 0214/15 to a prescribing rate of 10.6% in 2016/17 among providers in the intervention groups.

A systematic review describing the outcomes of A&F on professional practice and healthcare outcomes (*N*= 140 studies) found that A&F may be more effective when it is provided in more than one way, and when it includes explicit targets and an action plan [26], concluding that the way A&F is provided influences its effectiveness. It has been suggested that informational feedback, such as the feedback provided in our intervention groups, helps to mitigate the barriers associated with clinician lack of knowledge [28]. While there have been several studies that examined the use of A&F to reduce unnecessary antibiotic prescribing [29, 30] and the use of antipsychotics in patients diagnosed with dementia [21, 30], these studies did not assess whether a differential effect was noted based on type of feedback provided. Additionally, we found that practice location and remuneration model of the provider was associated with prescribing rates and maybe an important consideration for future A&F strategies.

This study establishes that A&F interventions can be a viable means to reduce unnecessary care in this and other domains of primary care. The ubiquitous use of electronic medical records in primary care and the increasing capacity to use these data to improve care makes scaling up this type of intervention a viable means to improve care in similar complex practice areas. This study confirms that A&F interventions are useful in reducing potentially unnecessary care and thus reducing extraneous health care expenditures that do not lead to improved patient outcomes.

### Limitations

There are limitations of this study. Firstly, we do not know if providers in MaPCReN are representative of all primary care providers in Canada or internationally. Since participation in MaPCReN is voluntary it is possible that some of the change in prescribing reflects a selection bias towards those that are interested in practice improvement and research. There are provider, clinic and health system characteristics that were not available in the MaPCReN dataset that may have influenced the study results. This may have included race/ethnicity of the patient or provider, physician quality improvement knowledge and training, or clinic structure (i.e. health team vs. individual stand-alone practice). Additionally, we did find that there were differences in our clusters based on rurality, renumeration model and medical training location. Rurality and renumeration model were associated with prescribing reductions. Further research should explore the association between rurality and renumeration and prescribing rates.

## Conclusions

In conclusion, we found that both practice-specific and background information sent directly to primary care providers by a trusted source reduced potentially unnecessary prescriptions. Our findings support further scale up of efforts to engage with primary care practices to improve care with practice level data.

## Supplementary Information

**Additional file 1.**

## Data Availability

The datasets generated and/or analysed during the current study are not publicly available due to the confidential nature of data governed by the PHIA legislation but are available from the corresponding author if appropriate approvals are obtained.

## Abbreviations

A&F: Audit and Feedback
CWC: Choosing Wisely Canada
MaPCReN: Manitoba Primary Care Research Network
CPCSSN: Canadian Primary Care Sentential Surveillance Network
EMR: Electronic Medical Record
ATC: Anatomical Therapeutic Chemical code
ICD-9-CM: International Classification of Diseases, ninth revision, clinical modification
OR: Odds Ratio
CI: 95% Confidence Interval
