# Supplementary material for: Implementing an audit and feedback cycle to improve adherence to the Choosing Wisely Canada recommendations: clustered randomized trail
Source: BMC Prim Care. 2022 Nov 26;23:302. doi: 10.1186/s12875-022-01912-7 (PMC9701433; doi:10.1186/s12875-022-01912-7)

Appendix A: Standard Feedback Report
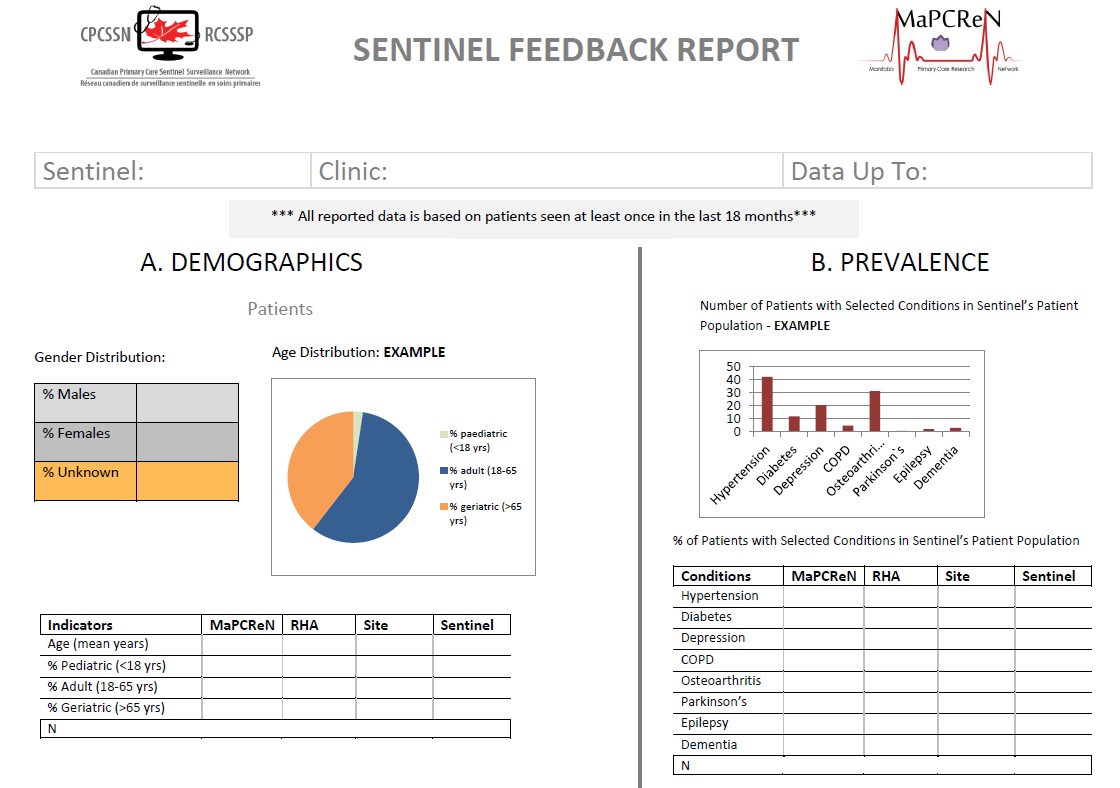


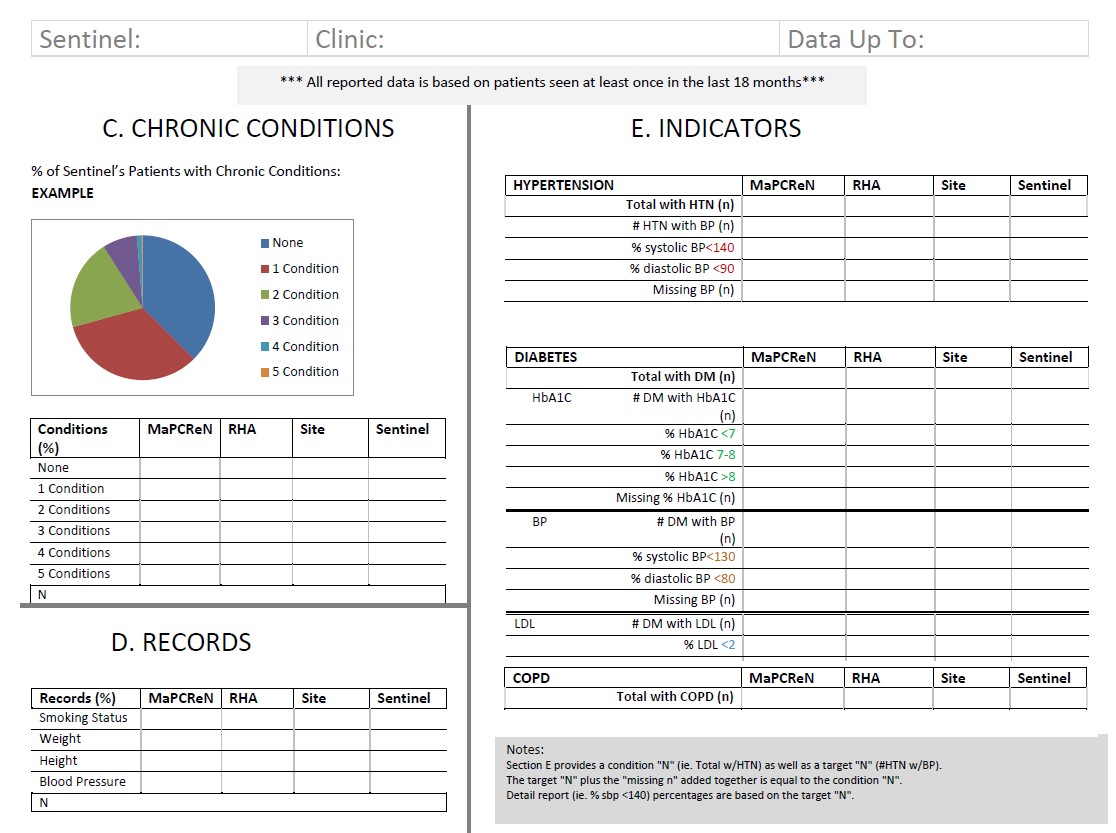


Appendix B: Choosing Wisely Canada Recommendations Summary


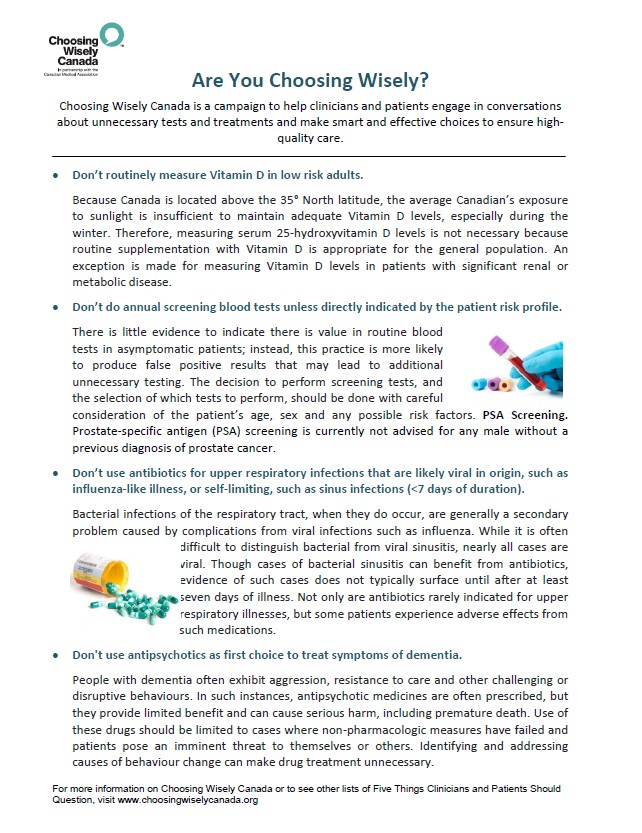


Appendix C: Modified Feedback Report


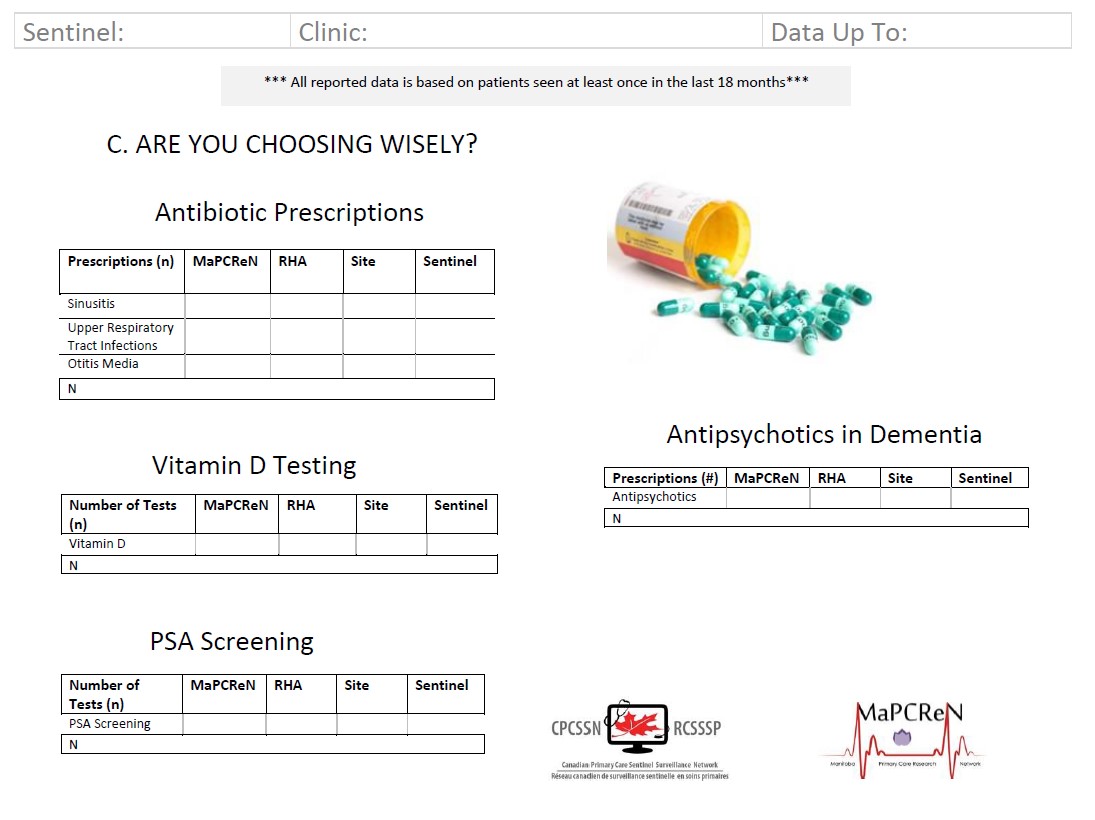

Supplement: Supplementary file 1 — Additional file 1. [file 12875_2022_1912_MOESM1_ESM.docx]
